# A new automated cell counter for mammalian cell culture assessment

**DOI:** 10.1186/1753-6561-9-S9-P51

**Published:** 2015-12-14

**Authors:** John P Carvell, Kathryn M Thomson

**Affiliations:** 1 Aber Instruments Ltd., Aberystwyth, SY23 3AH, UK

## Background

Performing accurate sample analyses during the culture of mammalian cells is vital in order to track the number and viability of cells present. This allows operators to recognise when reagents may need to be added to maximise yield, harvest cells appropriately and to identify when a targeted cell concentration has been reached. This is commonly performed via manual cell counts. Cell stains, such as Trypan blue are classically utilised for 'dye exclusion' assessment of viability, whereby dead cells are stained the colour of the dye and live cells remain colourless. However, this method is accompanied by many potential sources of human error and subjectivity as well as being a highly time consuming, labour-intensive mode of sample analysis. Various technologies introduced to the market in recent years automate this process in order to remove some human error and subjectivity associated with manual cell counts. However, the industry has not fully accepted these technologies, as the dye exclusion method is still the familiar and standard mode for assessing viability.

**Figure 1:**
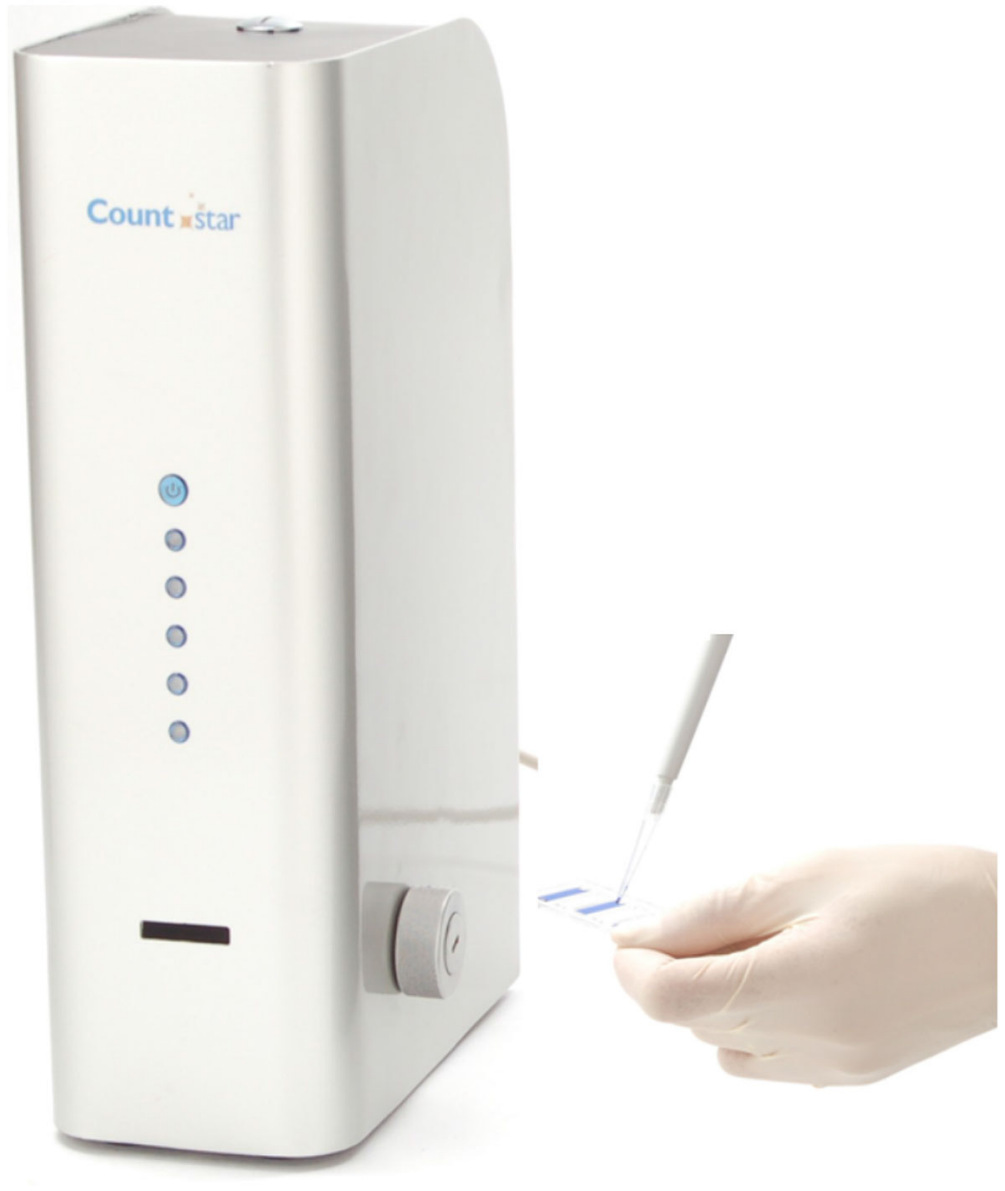
**The Countstar automated cell counter and disposable slide**.

This paper focuses on a new automated cell counter, the 'Countstar' as seen in figure 1, which uses brightfield image analysis and the trypan blue dye-exclusion method. This has the advantage of automating the process to remove human error and subjectivity, while retaining the standard trypan blue dye-exclusion method. The Countstar instrument uses individually packaged disposable plastic slides and associated software to analyse 20 μl samples. Each slide contains five separate chambers to lower costs and waste. Once a sample is loaded and the cells have settled, the instrument takes around 10 seconds to analyse an image. The Countstar requires no regular maintenance and will save vital time in the laboratory, while reducing human error associated with manual cell counts.

The Countstar provides users with the live cell count, % viability and average cell size. It also offers aggregate and cell size histograms and a circularity index, which can be used as an estimation of cell population health. The software also saves all images taken automatically for future reference and allows users to manipulate previously recorded data if required. In this paper, an in depth assessment of the Countstar is carried out and compared to manual cell counts for a range of mammalian cells.

## Materials and Methods

The software has been tested with a wide range of cells (approximately fifty cell lines), including CHO, myeloma, T cell and erythrocyte cell lines. A mixture of live, mixed and dead cultures were analysed using the Countstar to test the images produced. Further tests on the Countstar compared cell count and viability% results to manual cell counts for certain cell lines. This was first done by performing dilutions of a CHO cell culture to test how readings compared with those from a haemocytometer throughout a range of concentrations. Furthermore, five repeats were performed to compare deviation using the two methods at various dilutions. Finally, comparisons were made between cell counts using a single dilution for multiple cell lines using both the Countstar and haemocytometer, again with five repeat readings for each cell line.

## Results

The images produced by the Countstar were clear for all cell lines tested and the software was able to reliably detect changes in viability between live, dead and mixed in cultures that were analysed.

The averages for both cell count and viability produced by the Countstar were shown to be in line with results from a haemocytometer throughout the dilutions performed, as shown by high correlation values for the two methods (R2 = 0.9987; R2 = 0.9985 respectively). A smaller variation was seen between five repeat readings for each dilution with a CHO cell line with the Countstar than with the haemocytometer throughout the dilution series in regards to both cell count and viability%. This was also the case when single dilutions were tested for a range of cell lines, with the Countstar continuously displaying a lower deviation between repeats than manual counts for each cell line compared.

## Conclusions

The results demonstrate that the Countstar is able to accurately determine cell concentration and viability in line with manual cell counts throughout a wide concentration range. The tests demonstrate that the instrument is able to track decreases in viability, along with manual assessments for a cell culture. Smaller variation was observed between repeats using the Countstar when compared with haemocytometer readings for the range of dilutions analysed and for a range of cell lines tested at single dilutions. The results from these experiments show that the Countstar can reliably analyse a range of cell cultures and was shown to improve consistency between counts for a wide concentration range when compared to the use of the haemocytometer. The Countstar may be a good alternative to manual cell counting, as uses the same dye exclusion method and provides similar readings with a greater consistency in less time than manual counts.

